# An Interactive Web-Based Sexual Health Literacy Program for Safe Sex Practice for Female Chinese University Students: Multicenter Randomized Controlled Trial

**DOI:** 10.2196/22564

**Published:** 2021-03-12

**Authors:** Janet Yuen-Ha Wong, Wen Zhang, Yongda Wu, Edmond Pui Hang Choi, Herman Hay Ming Lo, Wendy Wong, Jasmine Hin Man Chio, Hau Lin Cherry Tam, Fei Wan Ngai, Marie Tarrant, Man Ping Wang, Hextan Yuen-Sheung Ngan, Daniel Yee-Tak Fong

**Affiliations:** 1 School of Nursing University of Hong Kong Hong Kong SAR Hong Kong; 2 Department of Applied Social Sciences The Hong Kong Polytechnic University Hong Kong SAR Hong Kong; 3 School of Chinese Medicine The Chinese University of Hong Kong Hong Kong SAR Hong Kong; 4 Department of Counselling and Psychology Hong Kong Shue Yan University Hong Kong SAR Hong Kong; 5 Department of Social and Behavioural Science City University of Hong Kong Hong Kong SAR Hong Kong; 6 School of Nursing The Hong Kong Polytechnic University Hong Kong SAR Hong Kong; 7 School of Nursing The University of British Columbia Vancouver, BC Canada; 8 Department of Obstetrics and Gynaecology University of Hong Kong Hong Kong SAR Hong Kong

**Keywords:** sexual health, eHealth, women's health, sex education, health literacy

## Abstract

**Background:**

Sexual health concerns among young adults worldwide help to motivate preventative practices against sexually transmitted infections. To foster better sexual health, sexual health literacy must be enhanced. Little research has been conducted on the impact of gender power dynamics on sexual health, such as sexual coercion, even though the prevalence of sexual coercion remains high in China.

**Objective:**

This study describes the development and systematic evaluation of a web-based sexual health literacy intervention called “Smart Girlfriend” for female Chinese university students.

**Methods:**

A multicenter randomized controlled trial was conducted with 781 female university students at 5 universities with dormitories in Hong Kong. Inclusion criteria were used to select unmarried, female, Chinese university students who were ≥18 years old and had not received a sexual health intervention in the past 12 months. Participants were randomly assigned to 2 groups: one group received an interactive web-based sexual health literacy intervention and the other group received a single webpage of online information about condom use. The intervention content was based on the Health Belief Model and the Continuum of Conflict and Control theory. The primary outcome was self-reported consistency of condom use with every partner at 3-month and 6-month follow-up assessments, analyzed using zero/one inflated beta (ZOIB) regression. The secondary outcome was an appraisal of the knowledge, attitudes, norms, and self-efficacy of condom use using the 25-item Multidimensional Condom Attitudes Scale (MCAS). The intention to treat was applied in analyses.

**Results:**

Of 1503 individuals that were screened, 781 (52%) were randomized into 2 groups. The retention rates at the 3-month and 6-month follow-ups were 92% and 91%, respectively. Most participants were born locally (536/746, 72%), and 18% (134/746) self-reported as a sexual minority. ZOIB results regarding the consistency of condom use were not significant [model 1: odds ratio (OR) 2.25 with a 95% credible interval (CrI) of 0.84-6.36; model 2: OR 8.03 (95% CrI 0.22-330.31); model 3: OR 1.21 (95% CrI 0.78-1.86)]. Consistency in the intervention group was 5% higher (95% CI −1.90 to 11.63) than the control group at the 3-month follow-up, and 1% higher (95% CI −5.81 to 8·02) at the 6-month follow-up. MCAS scores at the 3-month follow-up were significantly higher in the intervention group (mean 122.51, SD 15.97) than the control group (mean 119.86, SD 15.85; *P*=.02).

**Conclusions:**

An interactive web-based sexual health literacy program did not significantly increase the consistency of condom use compared to a single webpage of condom use information; however, it did temporarily improve knowledge, attitudes, norms, and self-efficacy regarding condom use. Future revisions of this intervention should be personalized and delivered with a proactive approach.

**Trial Registration:**

ClinicalTrials.gov NCT03695679; https://clinicaltrials.gov/ct2/show/NCT03695679

## Introduction

According to the World Health Organization (WHO), sexual health is a state of physical, mental, and social well-being in relation to sexuality [1]. Sexually healthy individuals have an absence of sexual or reproductive disease and a positive approach to managing respectful sexual relationships free of coercion and violence, thereby exhibiting safe sexual behaviors. Sexual health helps prevent sexually transmitted infections (STIs) and the human papillomavirus (HPV), the second leading cause of cancer deaths among women globally [2], which the WHO proposed as a key global health sector strategy for 2016-2021 [3].

Previous studies have indicated the important role of health literacy in promoting sexual health [4-6]. Health literacy is a form of empowerment to enhance individuals' capacity to access, understand, communicate, and process health information and services to make appropriate health decisions [7]. Sexual health literacy is the ability to understand preventive sexual health information to make informed choices, increase safe sex practices (eg, promoting condom use, limiting the number of sexual partners, avoiding causal sex, and enhancing sexual communication and negotiation skills with regard to sex refusal, condom use, and a partner's STI history), and reduce STI risk [4]. Low sexual health literacy is related to poor sexual health decision-making among university students, including engaging in risky sexual behaviors (such as inconsistent condom use) and delays or difficulties in seeking care [5]. Young adults might generally have good health and thus may not fully understand the importance of sexual health assessment in the absence of obvious symptoms, particularly after engaging in risky sexual behaviors [6]. Due to increasing trends of premarital sex and unsafe sexual behaviors [8], technologically advanced dating apps [9,10], and engagement in compensatory dating and casual sex [11], the risks of STIs and cervical cancer in female university students is high. Therefore, it is paramount that female university students have adequate sexual health literacy to facilitate safe sex practices, which yields better sexual health.

In this study, we operationalize sexual health literacy as individuals' capacity to understand risk information about unsafe sex practices and communicate with sexual partners to make optimal decisions and maintain sexual health. Previous sexual health literacy interventions focused on specific groups of people, such as a 50-minute face-to-face interactive class for HIV-positive people [12] and a 10-hour face-to-face intervention for jailed females to prevent cervical cancer [13]. However, few interventions targeted young females in the general population to prevent common STIs. Moreover, regarding the intervention content, it has been increasingly recognized that interventions should go beyond biophysical content, such as human development and contraception skills, by also including sociocultural content, such as respectful relationships and sexual coercion [14]. Research on effective interventions addressing sexual coercion and safe sex is limited, even though freedom from sexual coercion is a key conceptual component of good sexual health [15].

The internet was the most commonly accessed source for sexual health information in young adults [16]. A web-based sexual health intervention has some potential advantages over a traditional face-to-face intervention, including the capacity to reach a larger number of people in the population with a relatively low cost and facilitating communication with full privacy and confidentiality [16]. Moreover, a web-based intervention's interactive and anonymous delivery is more acceptable and effective [17]; a Cochrane meta-analysis evaluating 15 randomized controlled trials (RCTs) on safe-sex practice interventions found the interactive computer-based interventions to be more effective at improving knowledge about sexual health [18]. In this study, we have developed an interactive web-based sexual health literacy intervention to promote safe sex practices.

The sexual health status of young women in China is generally poor [19], and the level of sexual health knowledge was poorer than what was reported for young women in Western countries [20]. Young women are also vulnerable to risky sexual behaviors [21]. A national survey found that 1.6% of female Chinese university students reported having multiple sexual partners [22], and another study found that only 17.2% of sexually active female Chinese university students consistently used condoms [23]. The prevalence of sexual coercion of female university students in Hong Kong was reported to be approximately 13% in 2015, showing no reduction since 2008 [24,25]. Sexual health interventions in Hong Kong have lagged far behind that of many other places [26]. Moreover, sexual health interventions in Hong Kong often focus primarily on physiological knowledge and the dissemination of STI and STI-prevention information; little attention is paid to the effects of gender-power dynamics on sexual health, such as sexual coercion, respectful relationships, and sexual communication and negotiation [26]. However, a growing body of literature shows that the more sexual communication and negotiation that occurs before sex, the more likely a condom will be used during sex [27]. Sexual coercion is highly related to unsafe sex practices [28]. Taken together, these facts emphasize a need to revisit, develop, and evaluate comprehensive sexual health literacy interventions in the Chinese context.

In this study, we describe the development and systematic evaluation of a sexual health literacy program called “Smart Girlfriend” using a multicenter RCT. This program is an interactive web-based intervention that aims to disseminate knowledge about STIs and condom use, communication and negotiation about condom use, and sexual coercion in daily life to enhance safe sex practices among female university students in Hong Kong.

## Methods

### Trial Design

The design was a multicenter, single-blind, parallel-group RCT, registered with ClinicalTrials.gov (NCT03695679). Ethical approval was obtained from the institutional review board of the University of Hong Kong/Hospital Authority Hong Kong West Cluster (UW-17029) and other universities. We followed the CONSORT (Consolidated Standards of Reporting Trials) guidelines.

### Sample Size

Our sample size calculation was based on a primary comparison of behavioral change in the consistency with condom use. A previous study reported that the mean percentage of condom use was 68% (SD 39%) in individuals who underwent a computer-based intervention, compared with 24% (SD 35%) among those in a control condition, corresponding to a moderate to large Cohen effect size of 0.6 [29]. To conservatively detect a small Cohen effect size of 0.3 with 80% power and a maximum 5% false positive error rate using a 2-sided, 2-sample *t* test, a total of 352 (ie, 2 groups of 176) female university students were required. Assuming a 30% attrition rate based on a previous study using a web-based intervention [30], we calculated a total necessary sample size of 500 participants.

### Participants

In total, 1503 female university students across various disciplines and years of study were screened from 5 universities with dormitory or residential halls in Hong Kong. Of these, 781 students were recruited. The eligibility criteria were (1) female university students aged 18 years or above who (2) are able to read Chinese or understand Cantonese, (3) are unmarried, (4) have been with intimate partners (ie, current and former dating partners or partners in a relationship) in the past 12 months, and (5) have not received any sexual health information (including formal face-to-face or online education or training courses related to contraceptives and sexually transmitted diseases, from a university, hospital, clinic, or nongovernmental organization) in the past 12 months. The exclusion criteria were (1) an unwillingness to complete the questionnaires at 3 time-points, (2) being pregnant or postnatal, and (3) having a psychiatric illness.

### Recruitment, Randomization, Masking, and Allocation Concealment

Students were reached via leaflets, campus booths, bulk emails, and posters. All interested participants received an invitation email to log into the Smart Girlfriend webpage (Multimedia Appendix 1-3). Online enrolment was used to screen students for eligibility. For eligible participants, written informed consent was obtained online, followed by a baseline questionnaire. After completing the questionnaire, the recruited participants were randomized to either the intervention group or the control group, according to a list prepared by blocked randomization (with blocks of 4), with a 1:1 randomization ratio. The block size and order of allocation were kept securely in the randomizer to avoid selection bias. The online platform conducted masking and allocation concealment according to the participants' enrolment sequence. Participants were automatically guided to the webpage associated with their allocation and were not aware of the group allocation in advance. Participants who completed all of the web-based questionnaires were given vouchers with a value of HKD $300 (USD $38.50). The privacy of all participants was ensured. Data collected from all questionnaires were stored in a protected university database.

### Previous Development Efforts of Smart Girlfriend

Findings from our previous local multisite survey (n=1076) revealed that Chinese university students saw the relationship between unsafe sex practices and sexual coercion as problematic [25]. The results showed that 12% of female university students who were engaged in a dating relationship or had dating partners in the past year experienced sexual coercion within that year [25]. Therefore, we conducted an intervention to help enhance the awareness of sexual coercion among university students. Sexual issues are typically taboo subjects in Chinese culture; therefore, to minimize embarrassing situations for participants, we implemented the Dating Compassion, Assessment, reFerral, and Education (Dating CAFE) Ambassador Programme to help Chinese university students with dating violence. The intervention's development was based on the theory of planned behaviors and was conducted via 3 face-to-face workshops (totaling 7.5 hours) with 81 university students. Compared to students in the control group, we found that the students trained to be ambassadors had significantly enhanced behavioral intentions and control to help peers who were experiencing dating violence, decreased acceptance of dating violence, and increased subjective norms for helping others [31]. Moreover, we learned that discussions about dating issues were attractive and acceptable among university students. In addition, the face-to-face intervention was labor-intensive, and some students could not be enrolled due to timetable clashes. Thus, a web-based intervention could be a more cost-effective and practical approach for reaching as many eligible young people as possible.

### The Smart Girlfriend Intervention

**Figure 1:**
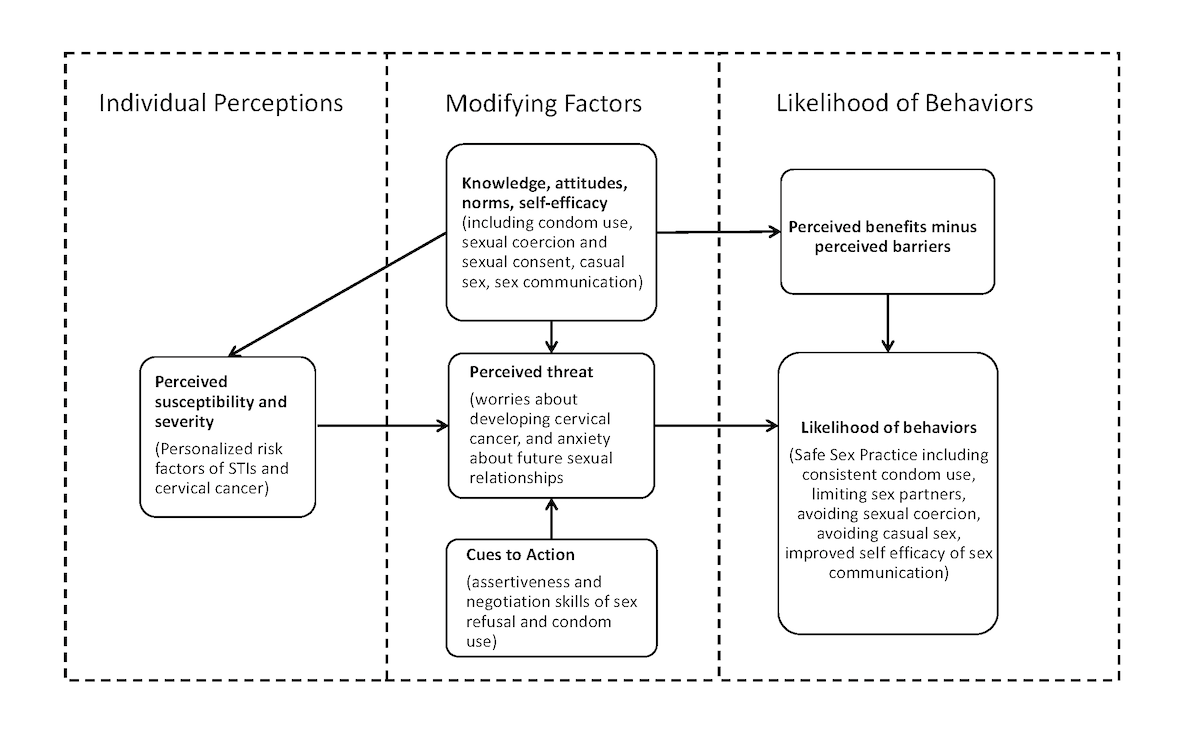
Conceptual framework of the Smart Girlfriend program based on the Health Belief Model. STIs: sexually transmitted infections.

**Figure 2:**
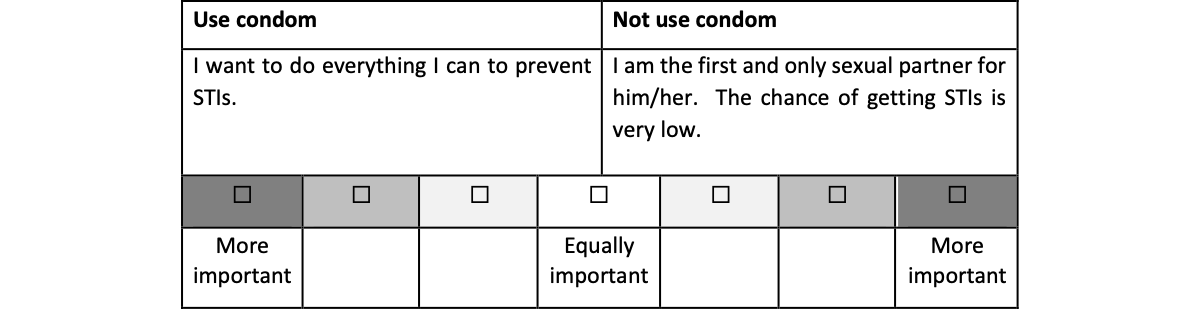
An example of a scale-based question asked by Smart Girlfriend to help students think about the positive and negative features of condom use and provide an opportunity for feedback and reflection on what matters most to them. STIs: sexually transmitted infections.

The development of Smart Girlfriend was based on the Dating CAFE initiative. Smart Girlfriend is designed to be a sexual health literacy intervention empowering female university students with enhanced knowledge, attitudes, norms, and self-efficacy around managing sexual health, particularly condom use for safe sex practice. Based on the Health Belief Model (Figure 1) [32], we delivered Smart Girlfriend in 3 phases. In the first phase, participants were able to check their perceived susceptibility and perceived severity for individual STIs. The Harvard Cancer Risk Index [33] was used to ask 8 questions to estimate individual STI and cervical cancer risks. Respondents obtained personalized results from their answers, including factors that may increase or reduce their risk of getting STIs and cervical cancer. In the second phase, participants were given knowledge-based information about STIs and cervical cancer in text format, including relevant statistics, development, possible symptoms, and prevention methods. Some scale-based questions were asked to help the students think about the positive and negative features of condom use. Participants were able to offer personal feedback and were provided with an opportunity to reflect on which benefits or barriers mattered most to them (Figure 2).

In the second phase, participants were also prompted to take action regarding condom use. Therefore, condom use procedures and tips, as well as web links for local STI testing, cervical screening programs, and HPV vaccination programs, were provided. In addition, participants' self-efficacy in condom use was enhanced by providing information about assertiveness in sexual consent and sexual communication to avoid sexual coercion and casual sex. Three 5-minute videos were created with narrative stories about STIs and HPV infection based on different scenarios relevant to common situations for university students, about the handling of sexual consent in dormitories, engaging in sexual communication at home, and talking to a friend about worries regarding sex without a condom after a Christmas party (Multimedia Appendix 2). The Continuum of Conflict and Control (CCC) theory [34] was used to guide the stories, emphasizing that sexual coercion can occur without physical violence and with minimal fear to strengthen participants' knowledge about sexual coercion, sexual consent pertaining to condom use, and sex refusal. Participants were able to assess their own values and receive feedback on their choices. Textbox 1 shows the perceived benefits of and barriers to condom use featured in the interactive intervention.

**Perceived benefits of and barriers to condom use featured in the interactive intervention.**

**Perceived benefits of condom use:**

- Consistent use of latex condoms before having sex lowers the risk of cervical cancer.
- Consistent and correct condom use is necessary every time one has sex, from the start of the act to the finish, to effectively lower the risk of sexually transmitted infections (STIs).
- A condom acts as a barrier against human papillomavirus (HPV) and other STIs.
- Some types of HPV can cause cells in the cervix to become cancerous; a condom acts as a barrier against HPV and other STIs.

**Perceived barriers to condom use:**

- Not knowing the risk of STIs [Link to knowledge about STIs and cervical cancer, including its statistics, development, and possible symptoms]
- Not knowing how to use a condom [Link to condom use procedures and tips]
- Low comfort levels with initiating conversations about condom use
- Feelings of frustration when being rejected due to issues around condom use [Link to video 3 about feelings and provide information to connect condom use and health risks of STIs and cervical cancer]
- Not knowing what to do after being rejected due to issues around condom use
- Sexual coercion [Link to definition and statistics of sexual coercion and examples]

In the last phase, a page designed to summarize the participants' individual factors for facilitating decision-making about consistent condom use in future sexual activities was presented. Participants were asked to rate their level of self-efficacy in terms of knowledge, skills, clarity of information, and perceived support and advice on a scale of 1-5. If the participant's level of self-efficacy was low (1-3), they were directed to relevant information via hyperlinks.

In the control group, participants received minimal intervention, with only a single webpage of online information about procedures and tips for condom use. The site for the control group had a similar graphic design to the one used for the intervention group, but no self-assessment material or online quiz questions were presented.

An inquiry system was created to handle questions from the participants. This system was designed to help participants in both the intervention and control groups if they needed any support or wanted to seek further clarification.

The time spent engaging with the online information was approximately 30 minutes for the intervention group and 10 minutes for the control group.

### Outcomes and Follow-up

All data were collected from the webpage. Participants were sent a reminder email and SMS text message for completing the online questionnaires at 3 time-points: baseline (T1); 3-months postintervention (T2); and 6-months postintervention (T3). The primary outcomes were the consistency of condom use with every partner, in accordance with the recommended guidelines of a systematic review of 56 studies [35], which used the percentage of male condom protected sex with every partner during the past 3 months.

Secondary outcomes were (1) knowledge, attitudes, norms, and self-efficacy of condom use, as appraised by the 25-item Multidimensional Condom Attitudes Scale (MCAS) [36]; (2) knowledge, attitudes, norms, and self-efficacy of sexual coercion and sexual consent, as measured by the 39-item Sexual Consent Scale–Revised (SCS-R) [37]; and (3) self-efficacy in sexual communication, estimated by the 20-item Sexual Communication Self-efficacy Scale (SCSES) [38]. The MCAS items were answered using a 7-point Likert scale, and total scores ranged from 7 to 175; a previous study has shown acceptable validity and reliability in the Chinese population [39]. The Cronbach alpha in this study was .84. The SCS-R contained 3 attitudinal subscales (positive attitude toward establishing consent, lack of perceived behavioral control, and sexual consent norms) and 2 behavioral subscales (indirect consent behaviors, and awareness and discussion). The SCS-R items were answered using a 7-point Likert scale, and the Cronbach alpha in this study was .67. The SCSES items were answered using a 4-point Likert scale, and total scores ranged from 20 to 80. With the exception of one of the SCS-R subscales (lack of perceived behavioral control), high scores on the scales indicated a high level of measured outcomes. The Cronbach alpha in this study was .94.

Other outcomes included participant inquiries, participant satisfaction, and participation in the intervention. Participants' inquiries were collected to understand their further needs. Satisfaction with the intervention was evaluated by recording the overall satisfaction of the intervention on a scale of 0-10. In addition, participants were asked which part of the intervention was most memorable. The higher the score, the higher the overall satisfaction with the intervention. The total recorded views of each website page (recorded via Google Analytics) and the incidence of searching for more information were measured.

Requested demographic information included sexual orientation, birthplace, dating relationship status, and history of childhood sexual coercion. Information regarding individual risk of STIs and cervical cancer (Multimedia Appendix 3) was collected at baseline, including age, age at first sexual intercourse experience, number of sexual partners during one's lifetime, frequency of condom use, history of being diagnosed with an STI, smoking status, history of giving birth, and history of having a Pap smear test. The experience of sexual coercion was measured using a 7-item subscale of the Revised Conflict Tactic Scale [40], which indicated whether it had happened and how often the behavior had occurred in the past year.

### Statistical Analysis

The primary outcome, consistency of condom use, was analyzed using a zero/one inflated beta (ZOIB) regression model because the raw data of the consistency of condom use were not normally distributed, exhibiting excessive zeros and ones (ie, consistency of condom use=100%). ZOIB is based on a piecewise distribution, which accounts for the probability mass at 0 or 1 and the probability density within (0,1). Bayesian-based results were obtained using Stan (Stan Development Team; 4 chains, 4000 iterations, 1500 warm-ups) [41]. Odds ratios (ORs) and credible interval (CrI) values were calculated for Bayesian-based analysis.

We adopted the intention-to-treat principle, and all study subjects were included in the analysis. Missing values at the 3- and 6-month follow-ups were replaced by the last observed value. Missing values at baseline were replaced by values from the 3- or 6-month follow-up. If there was no value obtained at any of the 3 time-points, the participant was excluded from the analysis. For other outcomes, the *t* test was applied for continuous data and the chi-square test was applied for categorical data. All P values were 2-sided, and P<.05 was considered statistically significant. R software (version 3.6.1; R Core Team) with “tidyr” and “brms” packages was used to analyze the data. Questions were downloaded from the inquiry system. Content analysis was performed to categorize the collected responses.

## Results

### CONSORT Flowchart

**Figure 3:**
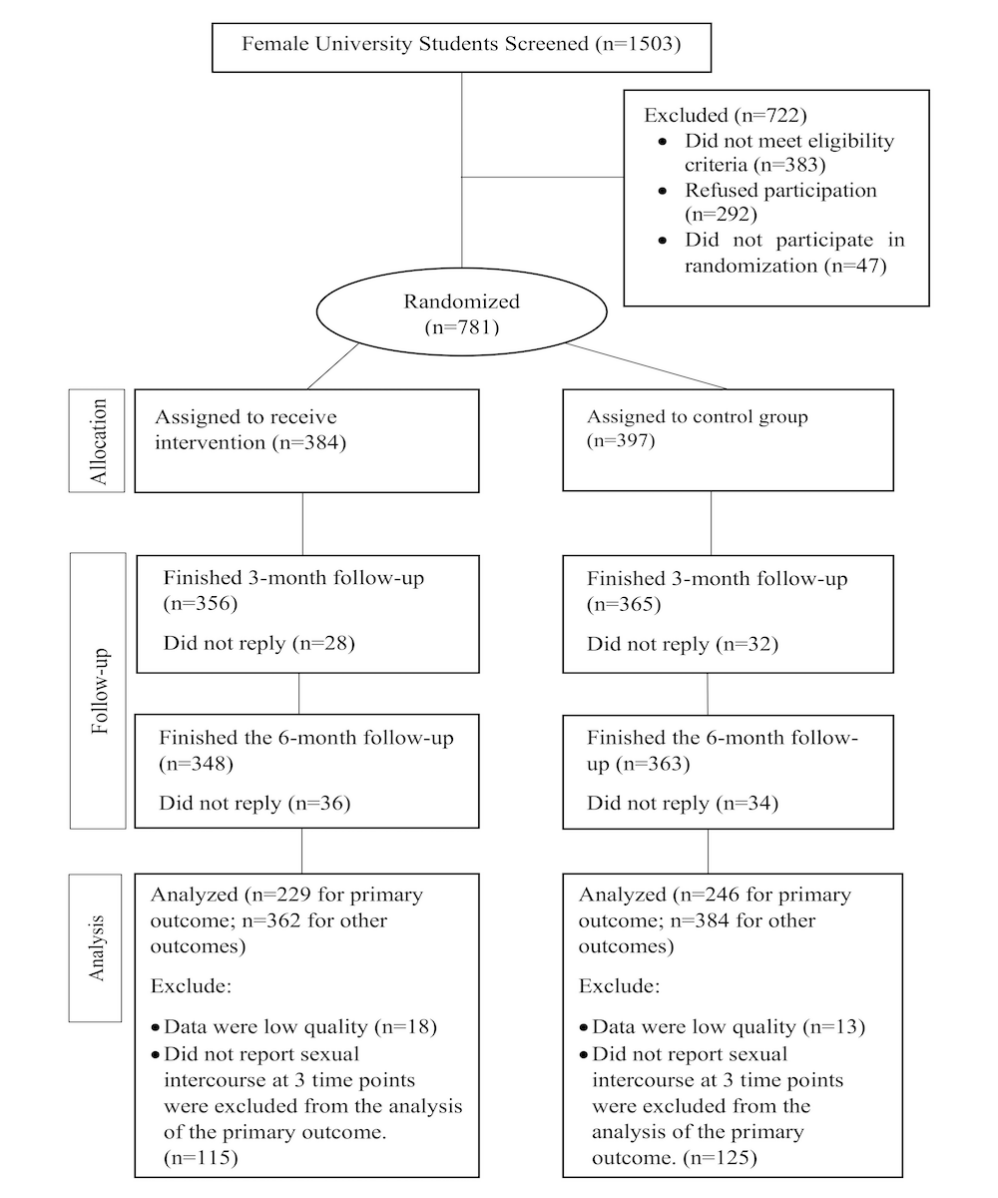
CONSORT flow diagram.

Figure 3 shows the flow of participants at baseline, grouping randomization, 3-month follow-up, and 6-month follow-up. Of 1503 screened students, 722 were excluded and 781 students were enrolled (enrolment rate: 52%), randomized into the intervention group (384/781, 49%) and the control group (397/781, 51%). Of the 781 included participants, the dropout rate was 8% (60/781) at the 3-month follow-up (intervention: 28/384, 7%; control: 32/397, 8%) and 9% (70/781) at the 6-month follow-up (intervention: 36/384, 9%; control: 34/397, 9%). Finally, 229 participants in the intervention group and 246 participants in the control group were included in the analysis of the primary outcome. A total of 362 participants in the intervention group and 384 participants in the control group were analyzed for other outcomes. Details of the quality check criteria used are provided in Multimedia Appendix 4.

### Baseline Characteristics of Participants

The mean age of participants was 21.5 (SD 2.6) years, ranging from 18 to 32 years. Most participants were born locally (536/746, 72%), and 18% (134/746) self-reported as sexual minorities (Table 1). Approximately 10% (78/746) of participants reported child sexual abuse experiences, and 22% (164/746) reported a history of sexual coercion.

**Table 1:** Characteristics of participants at baseline (n=746).

Characteristics		Total participants (n=746)	Intervention group (n=362)	Control group (n=384)
Age in years, mean (SD)		21.5 (2.6)	21.5 (2.7)	21.5 (2.6)
**Birthplace, n (%)**				
	Hong Kong	536 (72)	252 (70)	284 (74)
	Other	210 (28)	110 (30)	100 (26)
**Smoking status, n (%)**				
	Do not smoke	674 (90)	329 (91)	345 (90)
	Quit smoking	65 (9)	29 (8)	36 (9)
	Engages in smoking	7 (1)	4 (1)	3 (1)
**Alcohol consumption status, n (%)**				
	Do not consume alcohol	309 (42)	150 (41)	159 (42)
	Quit consuming alcohol	129 (17)	58 (16)	71 (19)
	Consumes alcohol	308 (41)	154 (43)	154 (40)
**Sexual orientation, n (%)**				
	Heterosexual^a^	612 (82)	294 (81)	318 (83)
	Sexual minority^b^	134 (18)	68 (20)	66 (17)
**History of child sexual abuse, n (%)**				
	Occurred	668 (90)	323 (89)	345 (90)
	Did not occur	78 (10)	39 (11)	39 (10)
**History of sexual coercion, n (%)**				
	Occurred	164 (22)	77 (21)	87 (23)
	Did not occur	580 (78)	285 (79)	295 (77)
**History of sexually transmitted infections, n (%)**				
	No	741 (99)	358 (99)	383 (99)
	Yes	5 (1)	4 (1)	1 (1)
**History of Pap smear test, n (%)**				
	Has not had a Pap smear test	702 (94)	339 (94)	363 (95)
	Has had a Pap smear test	44 (6)	23 (6)	21 (6)
**Relationship status, n (%)**				
	Dating/in a relationship	597 (80)	280 (77)	317 (83)
	Broken up/ cohabiting	149 (20)	82 (23)	67 (17)
**Sexual experience, n (%)**				
	Has not had a sexual experience with a partner	293 (39)	141 (39)	152 (40)
	Has had a sexual experience with a partner	453 (61)	221 (61)	232 (60)
Age of first sexual intercourse experience, in years (n=463^c^), mean (SD)		19.6 (2.5)	19.6 (2.5)	19.7 (2.6)
Number of sexual partners (n=463^c^), mean (SD)		2.1 (2.8)	2.2 (3.3)	2.0 (2.1)

^a^For the purposes of this study, *heterosexual* was defined as an individual who is exclusively attracted to the opposite sex.

^b^For the purposes of this study, due to small respective sample sizes, the category of *sexual minority* included all respondents who reported that their sexual orientation is “mostly heterosexual,” “bisexual,” “mostly homosexual,” “completely homosexual,” and “not sure.”

^c^This item only includes respondents who reported sexual experience at baseline.

### Outcomes

#### Consistency of Condom Use

ZOIB modeling revealed nonsignificant differences between the intervention and control groups in all models regarding the consistency of condom use (Table 2). The intervention group showed a nonsignificant trend toward being more likely to report 0% or 100% consistency of condom use compared to the control group (OR 2.25, 95% CrI 0.84-6.36), and of those, a nonsignificant trend toward being more likely to report 100% condom use consistency compared to the control group (OR 8.03, 95% CrI 0.22-330.31). At baseline, the consistency of condom use was 78% in the intervention group and 72% in the control group (95% CI −0.97 to 13.04). At the 3-month follow-up, the intervention group exhibited 5% higher consistency (intervention: 80% vs control: 75%, 95% CI −1.90 to 11.63) than the control group, and exhibited 1% higher consistency (intervention: 77% vs control: 76%, 95% CI −5.1 to 8.02) at the 6-month follow-up.

**Table 2:** Impact of the Smart Girlfriend program on the consistency of condom use at different time points.

Model, group, and time-point		Estimate (SE^a^)	OR^b^	95% CrI^c^
**Model 1: Report 0% or 100% condom use consistency in all participants**				
	Group	0.81 (0.52)	2.25	0.84-6.36
	Time3M^d^	0.06 (0.33)	1.06	0.56-2.02
	Time6M^e^	0.17 (0.33)	1.18	0.62-2.25
	Group* Time3M^f^	0.00 (0.48)	1.00	0.39-2.58
	Group* Time6M^g^	–0.04 (0.49)	0.96	0.37-2.48
**Model 2: Report 100% condom use consistency in those reporting 0% or 100%**				
	Group	2.08 (1.87)	8.03	0.22-330.31
	Time3M	0.85 (0.73)	2.35	0.58-10.55
	Time6M	1.18 (0.74)	3.26	0.80-14.91
	Group* Time3M	–0.66 (1.12)	0.52	0.06-4.57
	Group* Time6M	–2.66 (1.15)	0.07	0.006-0.62
**Model 3: Report a condom use consistency between 0% and 100% in all participants**				
	Group	0.19 (0.22)	1.21	0.78-1.86
	Time3M	–0.00 (0.15)	1.00	0.74-1.34
	Time6M	–0.04 (0.16)	0.96	0.70-1.31
	Group* Time3M	0.07 (0.24)	1.07	0.67-1.71
	Group* Time6M	0.01 (0.25)	1.01	0.63-1.67

^a^SE: standard error.

^b^OR: odds ratio.

^c^CrI: credible interval for a Bayesian-based analysis.

^d^Time3M: the 3-month follow-up.

^e^Time6M: the 6-month follow-up.

^f^Group*Time3M: the interaction effect between the group and the 3-month follow-up.

^g^Group*Time6M: the interaction effect between the group and the 3-month follow-up.

#### Secondary Outcomes

The MCAS scores of the intervention group significantly increased at the 3-month follow-up compared to the control group (intervention: 122.51, SD 15.97, vs control: 119.86, SD 15.85; *P*=.02; Table 3). No significant difference was found in other secondary outcomes.

**Table 3:** The impact of the Smart Girlfriend program on secondary outcomes at different time points.

Outcome and time-point		Intervention group (n=362), mean (SD)	Control group (n=384), mean (SD)	*t* test (df^a^=744)	*P* value
**Multidimensional Condom Attitudes Scale (MCAS)**					
	Baseline	120.24 (SD 15.39)	118.60 (SD 16.13)	1.42	0.16
	3-month follow-up	122.51 (SD 15.97)	119.86 (SD 15.85)	2.27	0.02
	6-month follow-up	123.55 (SD 15.76)	121.80 (SD 16.75)	1.47	0.14
**Sexual Consent Scale–Revised (SCS-R) subscale 1^b^**					
	Baseline	32.76 (SD 12.16)	32.89 (SD 11.93)	–0.15	0.88
	3-month follow-up	31.31 (SD 11.77)	32.04 (SD 11.43)	­–0.87	0.39
	6-month follow-up	30.91 (SD 11.40)	30.68 (SD 11.54)	0.28	0.78
**SCS-R subscale 2^c^**					
	Baseline	59.43 (SD 10.44)	59.27 (SD 10.43)	0.22	0.83
	3-month follow-up	59.18 (SD 9.83)	58.22 (SD 9.93)	1.34	0.18
	6-month follow-up	58.82 (SD 9.45)	59.31 (SD 10.75)	­–0.67	0.51
**SCS-R subscale 3^d^**					
	Baseline	28.63 (SD 5.80)	28.03 (SD 5.90)	1.42	0.16
	3-month follow-up	27.80 (SD 5.64)	28.01 (SD 5.86)	–0.48	0.63
	6-month follow-up	27.92 (SD 5.50)	27.96 (SD 5.77)	–0.09	0.93
**SCS-R subscale 4^e^**					
	Baseline	34.64 (SD 6.25)	34.99 (SD 6.13)	–0.77	0.44
	3-month follow-up	34.21 (SD 6.21)	34.37 (SD 6.27)	–0.34	0.73
	6-month follow-up	33.88 (SD 6.27)	34.71 (SD 6.27)	–1.81	0.07
**SCS-R subscale 5^f^**					
	Baseline	16.04 (SD 5.31)	16.29 (SD 5.28)	0.65	0.51
	3-month follow-up	16.61 (SD 5.32)	16.22 (SD 5.08)	1.02	0.31
	6-month follow-up	17.26 (SD 5.02)	16.88 (SD 5.25)	1.02	0.31
**Sexual Communication Self-efficacy Scale (SCSE)**					
	Baseline	62.01 (SD 10.94)	60.54 (SD 11.26)	1.81	0.07
	3-month follow-up	63.41 (SD 11.09)	62.08 (SD 10.73)	1.66	0.10
	6-month follow-up	63.56 (SD 10.76)	62.80 (SD 10.77)	0.95	0.34

^a^df: degrees of freedom.

^b^SCS-R subscale 1: (lack of) perceived behavioral control.

^c^SCS-R subscale 2: positive attitude toward establishing consent.

^d^SCS-R subscale 3: indirect behavioral approach to consent.

^e^SCS-R subscale 4: sexual consent norms.

^f^SCS-R subscale 5: awareness and discussion.

### Inquiry System

Of the 781 participants, 10% (81/781) sent inquiries via the webpage. Of these, 27% (22/81) of inquiries asked about condoms and other contraceptive methods, 28% (23/81) asked about STIs, 14% (11/81) asked about sexual behaviors, and 31% (25/81) asked about suggestions for intervention, technical issues, or other issues (Table 4). A significant difference was found for condoms and other contraceptive methods (χ^2^=9.78, P=.002) and STIs (χ^2^=6.71, *P*=.01) between the 2 groups. Compared with the control group, participants in the intervention group were more likely to send inquiries about STIs (intervention: 18 vs control: 5), whereas intervention group participants were less likely to send inquiries about condoms and other contraceptive methods (intervention: 6 vs control: 16). In the category of STIs, 3 participants in the intervention group asked whether there is a risk of STIs and how to prevent STIs among women who have sex with women.

**Table 4:** Inquiries sent by participants in the intervention and control groups.

Inquired issue	Total (n=81), n (%)	Intervention group (n=45), n (%)	Control group (n=36), n (%)	χ^2^	*P* value
Condom and other contraceptive methods	22 (27)	6 (13)	16 (44)	9.78^a^	.002
Sexually transmitted infections	23 (28)	18 (40)	5 (14)	6.71^a^	.01
Sex life	11 (14)	8 (18)	3 (8)	1.52	.22
Technical issue	6 (7)	2 (5)	4 (12)	1.30	.25
Suggestions for the website	8 (10)	5 (11)	3 (8)	0.17	.68
Others	11 (14)	6 (13)	5 (14)	0.01	.94

^a^*P*<.01.

### Satisfaction and Participation of Participants

A slight but statistically nonsignificant difference in satisfaction (*t*_677_=0.15; *P*=.89) was found between the intervention (n=353) and control (n=326) groups, with average satisfaction scores of 6.19 (SD 2.75) and 6.22 (SD 2.78), respectively. Among participants who reported the type of content they remembered most (Table 5), almost half of the participants in the intervention group (48/118, 41%) and more than half of the participants in the control group (58/107, 54%) reported that content related to condoms was most memorable. In addition, more intervention group participants reported that content about sexual consent was the most memorable (32/118, 27%) than control group participants (21/107, 20%). There was a significant difference between the 2 groups for seeking out more information about safe sex practice at the 3-month follow-up (intervention: 120/334, 36% vs control: 92/353, 26%; χ^2^=7.83, *P*=.005) but not at the 6-month follow-up (intervention: 105/326, 32% vs control: 97/353, 28%; χ^2^=1.81, *P*=.18). Data from Google Analytics revealed that the most visited pages of the intervention were the inquiry page and the page with information about risk factors for cervical cancer.

**Table 5:** Content that participants reported to be the most memorable.

Most memorable content	Intervention group (n=118), n (%)	Control group (n=107), n (%)
Cervical cancer	4 (3.3)	0 (0)
Sexually transmitted infections	8 (6.8)	1 (0.9)
Sexual coercion	3 (2.5)	1 (0.9)
Condoms	48 (40.7)	58 (54.2)
Sexual consent	32 (27.2)	21 (19.7)
Other	23 (19.5)	26 (24.3)

## Discussion

### Principal Findings

This multicenter RCT in Hong Kong is the first study to examine the effects of an interactive web-based sexual health literacy program to promote safe sex practice among female Chinese university students. The consistency of condom use increased over time in both groups. The intervention group exhibited 5% higher consistency at the 3-month follow-up and 1% higher consistency at the 6-month follow-up compared to the control group; however, these differences were not statistically significant. Thus, the results did not reveal an effect from the intervention for increasing the consistency of condom use.

One of the possible explanations for the lack of positive effect may be due to the passive exposure of the intervention to our target audiences. In our intervention, the main components were delivered passively, including the knowledge about STIs, the narrative stories about STIs, and the introduction about sexual coercion. A meta-analysis for HIV-prevention interventions also revealed that participants in active interventions (such as client-tailored counseling and other activities to improve behavioral skills) reported a greater behavioral change improvement compared to passive interventions (such as messages to share procondom information and norms and to verbally model skills); and likewise, passive interventions in the intervention groups did not differ significantly from the control groups [42]. These findings show the positive effect of active interventions and suggest that increasing active components would be necessary for future research in promoting safe sex practice.

Another potential reason for the lack of positive effect could be associated with the nonpersonalized nature of the web-based intervention content. According to the inquiries we received from the participants, some participants asked if women who had sex with women were at risk for STIs, and some intervention content was not applicable to them. Previous studies found that personalized web-based interventions were more effective than nonpersonalized interventions, and the difference between the nonpersonalized intervention group and the blank control group was nonsignificant [43]. Thus, behavioral change may be improved if we tailor the intervention content to the participant's characteristics, such as sexual orientation.

Additionally, the imbalanced ceiling effect for 2 groups might also contribute to the insignificant intervention effect. The proportion of participants reporting 100% condom use at baseline in the intervention group was significantly higher than the control group (68% vs 59%, respectively; χ^2^=3.90, *P*=.048. Owing to the ceiling effect, the potential capacity of improvement in terms of condom use in the intervention group would be less than the control group, and the effect of intervention might be underestimated [44].

Increased MCAS scores at the 3-month follow-up indicate that the Smart Girlfriend program improved participants' knowledge, attitudes, norms, and self-efficacy of condom use temporarily; however, there was no significant long-term effect on behavior observed at the 6-month follow-up. These results indicate that the intervention could temporarily improve knowledge, attitudes, norms, and self-efficacy of condom use in young adults but cannot ultimately change their behaviors. These findings were in line with a previous study [45]. Starosta et al [46] found that a web-based intervention improved attitudes toward condoms but could not change condom use at the 3-month follow-up. Thus, it would seem that improvement occurs, but it would decrease over time if there is no further exposure in the intervention [47].

### Strengths and Limitations

The strengths of the study include the detailed development of the intervention based on theory and previous experience. The low dropout rate in this study indicates that university students welcomed the intervention and that it served the needs of the target population. With the participation of 5 universities in Hong Kong, the study included a diversity of student population characteristics, sexual orientations, and sexual coercion experiences. ZOIB was employed in this study, given the nonnormal distribution data with excessive zeros and ones. In promoting sexual health literacy, self-reported condom use is characterized by a substantial number of zeros or ones and discrete nonzero counts [48]. Excessive numbers of zeros or ones in this dependent variable make it difficult to fit the data using traditional methods, including ordinary least squares models and mixed linear models, which may yield biased estimates of the results. The beta distribution is considered a versatile function that fits a broad range of probability distribution shapes. This study may serve as a promising reference for future studies with similar problems. A high participation rate (91%) was reported in this study, indicating a high acceptability of digital interventions among university students and suggesting that a large number of university students can be reached at a very low cost.

This study has implications for future studies. Temporary improvement was observed in the knowledge, attitudes, norms, and self-efficacy of condom use, but not observed postintervention in behavioral change; this suggests that future research should more thoroughly consider novel methods that maintain the intervention effect and increase the behavioral change effect of web-based sexual health literacy. First, an active approach for intervention delivery may lead to the greatest increase in condom use. For example, virtual reality's effectiveness for behavioral skills training and practical exercises has been demonstrated in other research fields [49]. Second, the intervention content should be more personalized, especially for our target participants, before the intervention. More specific and advanced information (eg, whether nonpenetrative sex is a risk factor for contracting STIs), more customized content (eg, for women who have sex with women), and personal online counseling through the website might be needed. Third, more attention should be paid to the organization of the external links to other websites. We provided external hyperlinks to other websites to give participants more opportunities to explore other related information. In hindsight, providing external hyperlinks might not have been ideal since they may have distracted the participants from the intervention pathway and led them away from (and discouraged the return to) our website [50]. Recent research tested a web-based basic version alongside a version with added links to external resources, and it was found that the latter version was not effective [51]. The external links could be listed in the last (or a separate) page in future revised interventions.

Our study has several limitations. First, despite our relatively large sample of participants, compared with many other sexual health interventions, our study might lack power concerning the primary outcome for the consistency of condom use. The number of sexually active participants during the 6-month follow-up was relatively lower than expected. We included those who only reported sexual activities in one of 3 questionnaires and used a conservative approach that considered sexually inactive participants as exhibiting no change. This conservative approach might bias our results. Second, there is a potential influence of the Hawthorne effect. A systematic review found that the Hawthorne effect could influence participants' behavior in RCTs [52]. In our study, self-reported data were collected, and participants knew that the program was designed to increase their consistency of condom use. Third, we did not collect information about the duration of participation. However, due to the digital intervention design, all participants received information about the whole intervention before completing the questionnaires. Finally, our results were vulnerable to differential error because self-reported data were collected. The intervention group may have misreported the consistency of condom use to a greater extent than the control group at baseline (intervention: 78% vs control: 72%), thereby biasing the estimated intervention effect. There is no alternative to self-reporting in web-based trials because collecting biological data via web-based systems is unfeasible. Differential error could not be avoided; however, our trial was conducted under conditions that maximize accurate reporting, using an anonymous web-based questionnaire that assured participants of confidentiality with an absence of investigators.

### Conclusion

Among university students in Hong Kong, an interactive web-based sexual health literacy program resulted in a small but statistically nonsignificant increase in the consistency of condom use, as well as a significant and temporary increase in knowledge, attitudes, norms, and self-efficacy of condom use, but not in sexual coercion, sexual consent, or sexual communication, when compared with participants receiving only one webpage of condom use information. The number of participants at enrolment and the high participation rate highlight the need for sexual health literacy programs for young adults. Moreover, a future revision of this intervention should be personalized and delivered with an active approach.

## Appendix

Multimedia Appendix 1 Smart Girlfriend: an interactive web-based sexual health literacy program for safe sex practice in female Chinese university students.

Multimedia Appendix 2 Smart Girlfriend Screenshot: 5-minute videos with narrative stories about sexually transmitted infections (STIs) and human papillomavirus (HPV) infection.

Multimedia Appendix 3 Smart Girlfriend Screenshot: information collection of individual risk of sexually transmitted infections (STIs) and cervical cancer.

Multimedia Appendix 4 Details of the data quality check.

Multimedia Appendix 5 CONSORT-EHEALTH (V 1.6.1) checklist.

## Abbreviations

CrI: credible interval
Dating CAFE: Dating Compassion, Assessment, reFerral, and Education
HPV: human papillomavirus
MCAS: Multidimensional Condom Attitudes Scale
OR: odds ratio
RCT: randomized controlled trial
SCSES: SCSES: Sexual Communication Self-efficacy Scale
SCS-R: Sexual Consent Scale-Revised
STI: sexually transmitted infection
WHO: World Health Organization
ZOIB: zero/one inflated beta

## References

[1] World Health Organization (2006). Defining sexual health: Report of a technical consultation on sexual health, 28-31 January.

[2] Ferlay J, Shin H, Bray F, Forman D, Mathers C, Parkin DM (2010). Estimates of worldwide burden of cancer in 2008: GLOBOCAN 2008. Int J Cancer.

[3] World Health Organization Human papillomavirus (HPV) and cervical cancer. Sexually transmitted infections (STIs) 2016.

[4] Vamos CA, Thompson EL, Logan RG, Griner SB, Perrin KM, Merrell LK, Daley EM (2020). Exploring college students' sexual and reproductive health literacy. J Am Coll Health.

[5] Fortenberry JD, McFarlane MM, Hennessy M, Bull SS, Grimley DM, St Lawrence J, Stoner BP, VanDevanter N (2001). Relation of health literacy to gonorrhoea related care. Sex Transm Infect.

[6] Massey PM, Prelip M, Calimlim BM, Quiter ES, Glik DC (2012). Contextualizing an expanded definition of health literacy among adolescents in the health care setting. Health Educ Res.

[7] Nutbeam D (2008). The evolving concept of health literacy. Soc Sci Med.

[8] Yip PSF, Zhang H, Lam T, Lam KF, Lee AM, Chan J, Fan S (2013). Sex knowledge, attitudes, and high-risk sexual behaviors among unmarried youth in Hong Kong. BMC Public Health.

[9] Yeo TED, Ng YL (2016). Sexual risk behaviors among apps-using young men who have sex with men in Hong Kong. AIDS Care.

[10] Choi EPH, Wong JYH, Fong DYT (2016). The use of social networking applications of smartphone and associated sexual risks in lesbian, gay, bisexual, and transgender populations: a systematic review. AIDS Care.

[11] Lee TY, Shek DTL (2013). Compensated dating in Hong Kong: prevalence, psychosocial correlates, and relationships with other risky behaviors. J Pediatr Adolesc Gynecol.

[12] Robinson C, Graham J (2010). Perceived Internet health literacy of HIV-positive people through the provision of a computer and Internet health education intervention. Health Info Libr J.

[13] Ramaswamy M, Lee J, Wickliffe J, Allison M, Emerson A, Kelly PJ (2017). Impact of a brief intervention on cervical health literacy: A waitlist control study with jailed women. Prev Med Rep.

[14] Gonçalves TR, Faria ER, Carvalho FTD, Piccinini CA, Shoveller JA (2017). Behavioral interventions to promote condom use among women living with HIV: a systematic review update. Cad Saude Publica.

[15] Edwards WM, Coleman E (2004). Defining sexual health: a descriptive overview. Arch Sex Behav.

[16] Minichiello V, Rahman S, Dune T, Scott J, Dowsett G (2013). E-health: potential benefits and challenges in providing and accessing sexual health services. BMC Public Health.

[17] Baxter L, Egbert N, Ho E (2008). Everyday health communication experiences of college students. J Am Coll Health.

[18] Bailey JV, Murray E, Rait G, Mercer CH, Morris RW, Peacock R, Cassell J, Nazareth I (2010). Interactive computer-based interventions for sexual health promotion. Cochrane Database Syst Rev.

[19] Yu J (2012). Teenage sexual attitudes and behaviour in China: a literature review. Health Soc Care Community.

[20] Chen M, Liao Y, Liu J, Fang W, Hong N, Ye X, Li J, Tang Q, Pan W, Liao W (2016). Comparison of Sexual Knowledge, Attitude, and Behavior between Female Chinese College Students from Urban Areas and Rural Areas: A Hidden Challenge for HIV/AIDS Control in China. Biomed Res Int.

[21] Wang B, Davidson P (2006). Sex, lies, and videos in rural China: a qualitative study of women's sexual debut and risky sexual behavior. J Sex Res.

[22] Sun X, Liu X, Shi Y, Wang Y, Wang P, Chang C (2013). Determinants of risky sexual behavior and condom use among college students in China. AIDS Care.

[23] Ma Q, Ono-Kihara M, Cong L, Pan X, Xu G, Zamani S, Ravari SM, Kihara M (2009). Behavioral and psychosocial predictors of condom use among university students in Eastern China. AIDS Care.

[24] Chan KL, Straus MA (2008). Prevalence and Correlates of Physical Assault on Dating Partners. TOSSCIJ.

[25] Wong JYH, Choi EPH, Lo HHM, Wong W, Chio JHM, Choi AWM, Fong DYT (2019). Intimate Partner Sexual Violence and Mental Health Indicators Among Chinese Emerging Adults. J Interpers Violence.

[26] Leung H, Lin L (2019). Adolescent Sexual Risk Behavior in Hong Kong: Prevalence, Protective Factors, and Sex Education Programs. J Adolesc Health.

[27] Xiao Z, Li X, Lin D, Jiang S, Liu Y, Li S (2013). Sexual communication, safer sex self-efficacy, and condom use among young Chinese migrants in Beijing, China. AIDS Educ Prev.

[28] Agardh A, Odberg-Pettersson K, Ostergren P (2011). Experience of sexual coercion and risky sexual behavior among Ugandan university students. BMC Public Health.

[29] Kiene SM, Barta WD (2006). A brief individualized computer-delivered sexual risk reduction intervention increases HIV/AIDS preventive behavior. J Adolesc Health.

[30] Warmerdam L, van Straten A, Twisk J, Riper H, Cuijpers P (2008). Internet-based treatment for adults with depressive symptoms: randomized controlled trial. J Med Internet Res.

[31] Wong JY, Tang NR, Yau JH, Choi AW, Fong DY (2019). Dating CAFE Ambassador Programme: Chinese College Students to Help Peers in Dating Violence. Health Educ Behav.

[32] Janz NK, Becker MH (1984). The Health Belief Model: a decade later. Health Educ Q.

[33] Colditz GA, Atwood KA, Emmons K, Monson RR, Willett WC, Trichopoulos D, Hunter DJ (2000). Harvard report on cancer prevention volume 4: Harvard Cancer Risk Index. Risk Index Working Group, Harvard Center for Cancer Prevention. Cancer Causes Control.

[34] Carlson Rg, Dayle Jones K (2010). Continuum of Conflict and Control: A Conceptualization of Intimate Partner Violence Typologies. The Family Journal.

[35] Noar SM, Cole C, Carlyle K (2006). Condom use measurement in 56 studies of sexual risk behavior: review and recommendations. Arch Sex Behav.

[36] Helweg-Larsen M, Collins BE (1994). The UCLA Multidimensional Condom Attitudes Scale: documenting the complex determinants of condom use in college students. Health Psychol.

[37] Humphreys TP, Brousseau MM (2010). The sexual consent scale-revised: development, reliability, and preliminary validity. J Sex Res.

[38] Quinn-Nilas C, Milhausen RR, Breuer R, Bailey J, Pavlou M, DiClemente RJ, Wingood GM (2016). Validation of the Sexual Communication Self-Efficacy Scale. Health Educ Behav.

[39] Choi EPH, Fong DYT, Wong JYH (2020). The use of the Multidimensional Condom Attitude Scale in Chinese young adults. Health Qual Life Outcomes.

[40] Straus MA, Hamby SL, Boney-McCoy S, Sugarman DB (2016). The Revised Conflict Tactics Scales (CTS2). Journal of Family Issues.

[41] Bürkner P (2018). Advanced Bayesian Multilevel Modeling with the R Package brms. The R Journal.

[42] Albarracín D, Gillette JC, Earl AN, Glasman LR, Durantini MR, Ho M (2005). A test of major assumptions about behavior change: a comprehensive look at the effects of passive and active HIV-prevention interventions since the beginning of the epidemic. Psychol Bull.

[43] Mevissen FEF, Ruiter RAC, Meertens RM, Zimbile F, Schaalma HP (2011). Justify your love: testing an online STI-risk communication intervention designed to promote condom use and STI-testing. Psychol Health.

[44] McCambridge J, Kypri K, Elbourne D (2014). In randomization we trust? There are overlooked problems in experimenting with people in behavioral intervention trials. J Clin Epidemiol.

[45] Sun WH, Wong CKH, Wong WCW (2017). A Peer-Led, Social Media-Delivered, Safer Sex Intervention for Chinese College Students: Randomized Controlled Trial. J Med Internet Res.

[46] Starosta AJ, Cranston E, Earleywine M (2016). Safer sex in a digital world: A Web-based motivational enhancement intervention to increase condom use among college women. J Am Coll Health.

[47] Sales JM, DiClemente RJ, Davis TP, Sullivan S (2012). Exploring why young African American women do not change condom-use behavior following participation in an STI/HIV prevention intervention. Health Educ Res.

[48] Gupta R, Szczesniak RD, Macaluso M (2015). Modeling repeated count measures with excess zeros in an epidemiological study. Ann Epidemiol.

[49] Hadley W, Houck C, Brown LK, Spitalnick JS, Ferrer M, Barker D (2019). Moving Beyond Role-Play: Evaluating the Use of Virtual Reality to Teach Emotion Regulation for the Prevention of Adolescent Risk Behavior Within a Randomized Pilot Trial. J Pediatr Psychol.

[50] Huizingh EKRE, Hoekstra JC (2003). Why do consumers like websites?. J Target Meas Anal Mark.

[51] Peels DA, van Stralen MM, Bolman C, Golsteijn RHJ, de Vries H, Mudde AN, Lechner L (2014). The differentiated effectiveness of a printed versus a Web-based tailored physical activity intervention among adults aged over 50. Health Educ Res.

[52] McCambridge J, Witton J, Elbourne DR (2014). Systematic review of the Hawthorne effect: new concepts are needed to study research participation effects. J Clin Epidemiol.

