# Supplementary figures and images for: An Interactive Web-Based Sexual Health Literacy Program for Safe Sex Practice for Female Chinese University Students: Multicenter Randomized Controlled Trial
Source: J Med Internet Res. 2021 Mar 12;23(3):e22564. doi: 10.2196/22564 (PMC7998327; doi:10.2196/22564)

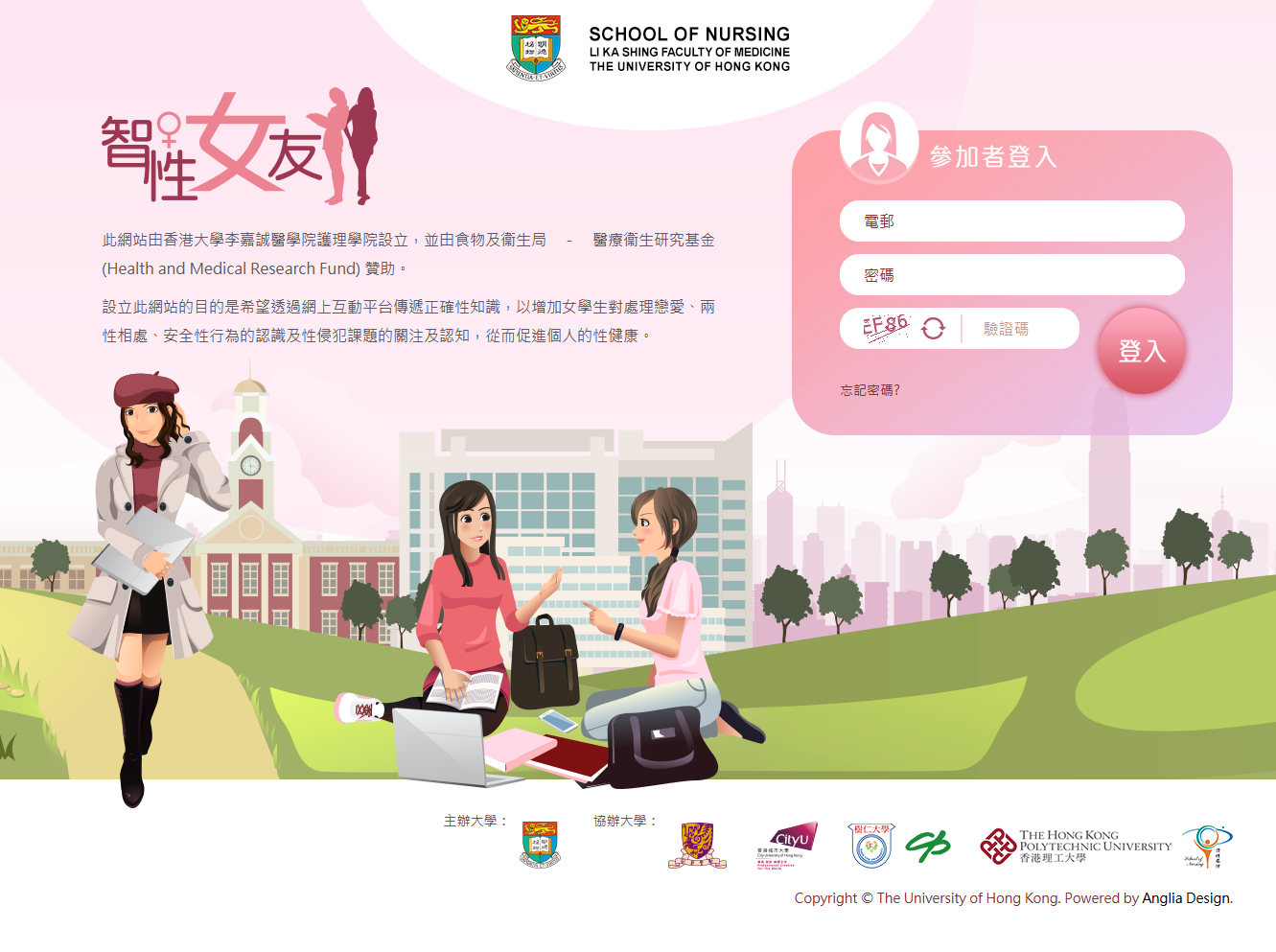

Supplement: Multimedia Appendix 1 [file jmir_v23i3e22564_app1.png]

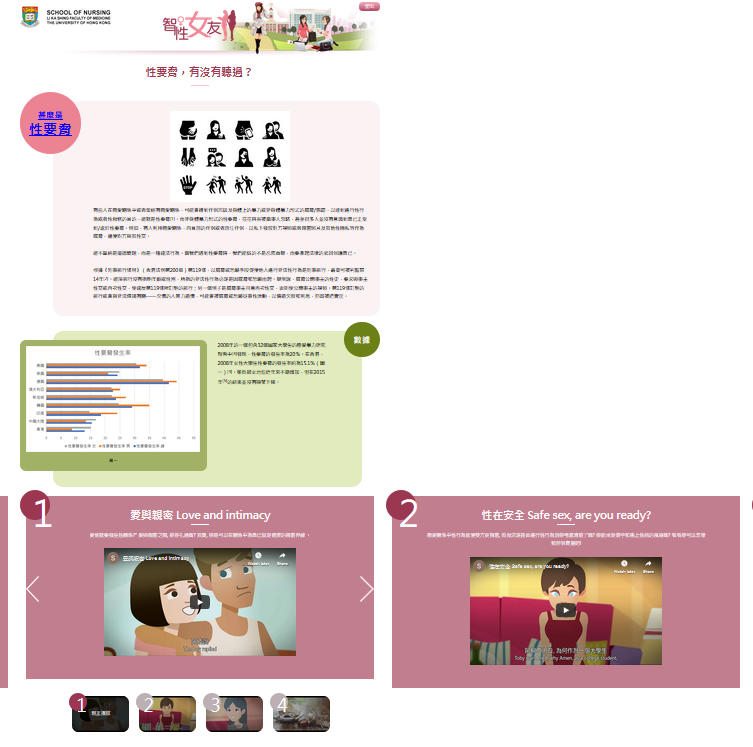

Supplement: Multimedia Appendix 2 [file jmir_v23i3e22564_app2.png]

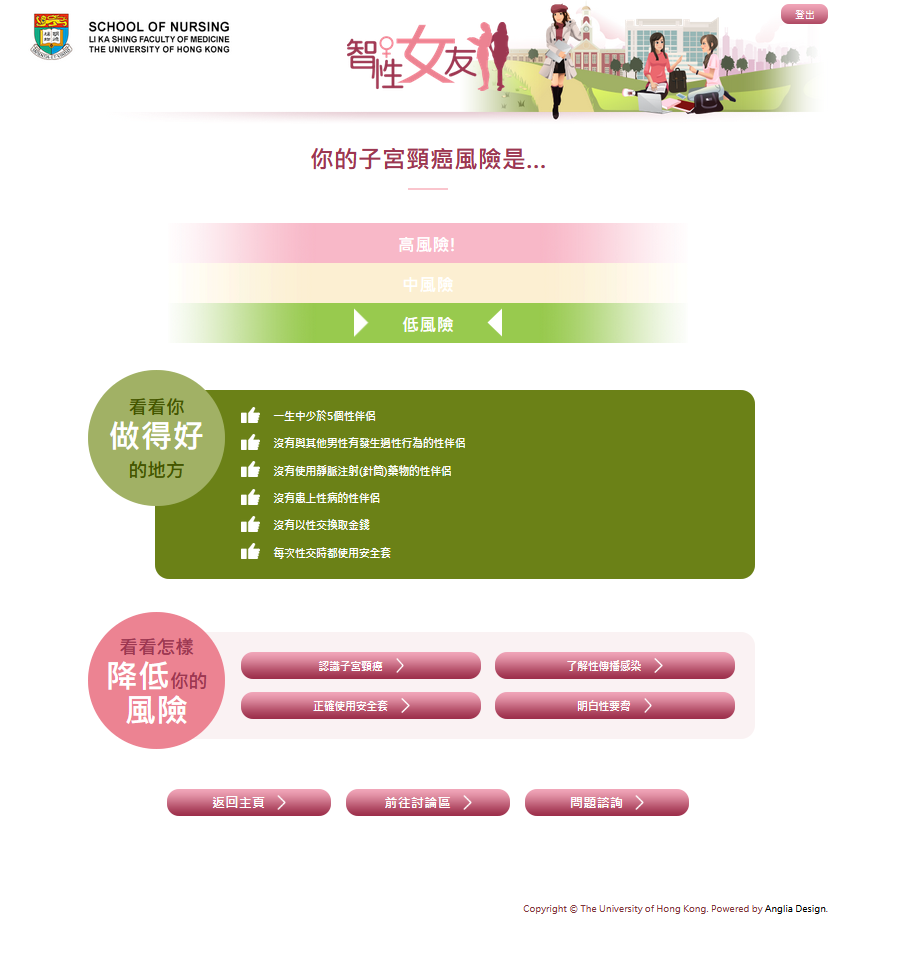

Supplement: Multimedia Appendix 3 [file jmir_v23i3e22564_app3.png]
