# Supplementary material for: An Interactive Web-Based Sexual Health Literacy Program for Safe Sex Practice for Female Chinese University Students: Multicenter Randomized Controlled Trial
Source: J Med Internet Res. 2021 Mar 12;23(3):e22564. doi: 10.2196/22564 (PMC7998327; doi:10.2196/22564)
Supplement: Multimedia Appendix 4 [file jmir_v23i3e22564_app4.docx]

Supplementary material

Details of checking the data:

a. Baseline Questionnaire

| Scenarios | Number of participants |
| --- | --- |
| 1.Drinking Status |  |
| (Use of alcohol before or during sexual intercourse in the past 3 months: Yes) AND (Drinking status: Never) | 0 |
| 2.Sexual Experience VS Sexual Experience |  |
| (sexual experience: No) AND (Experienced sexual coercion last year) | 21 (21/781=2.69%) |
| 3.Sexual Experience VS Number of Sexual Partners |  |
| (sexual experience: Yes) AND (number of sexual partners=0) | 6(6/781=0.7%) |
| (sexual experience: No) AND (number of sexual partners>0) | 0 |
| 4.Sexual Experience: NO AND Number of sexual partners in the past 3 months >0 | 0 |
| 5.Sexual Experience: NO AND Number of sexual intercourses in the past 3 months >0 | 1(1/781=0.13%) |
| 6.Sexual Experience: NO AND Number of condom use in the past 3 months >0 | 0 |
| 7.Sexual Experience VS alcohol and substance |  |
| sexual experience: NO AND use of alcohol before or during sexual intercourse in the past 3 months: YES | 2(2/781=0.26%) |
| sexual experience: NO AND use of substance before or during sexual intercourse in the past 3 months: YES | 0 |

b. 3-month follow-up (N=721)

| Scenarios | Number of participants |
| --- | --- |
| 1. Number of sexual partners in the past 3 months = 0 AND Number of sexual intercourses in the past 3 months >0 | 0 |
| 2. Number of sexual partners in the past 3 months = 0 AND Number of condom use in the past 3 months >0 | 1(1/721=0.14%) |
| 3. Number of sexual partners in the past 3 months = 0 AND use of alcohol before or during sexual intercourse in the past 3 months: YES | 3(3/721=0.42%) |
| 4. Number of sexual partners in the past 3 months = 0 AND use of substance before or during sexual intercourse in the past 3 months: YES | 0 |
| 5. Number of sexual intercourses in the past 3 months = 0 AND Number of condom use > 0 | 1(1/721=0.14%) |
| 6. Number of sexual intercourses in the past 3 months = 0 AND use of alcohol before or during sexual intercourse in the past 3 months: YES | 3(3/721=0.42%) |
| 7. Number of sexual intercourses in the past 3 months = 0 AND use of substance before or during sexual intercourse in the past 3 months: YES | 0 |

c. 6-month follow-up (N=711)

| Scenarios | Number of participants |
| --- | --- |
| 1. Number of sexual partners in the past 3 months = 0 AND Number of sexual intercourses in the past 3 months >0 | 0 |
| 2. Number of sexual partners in the past 3 months = 0 AND Number of condom use in the past 3 months >0 | 1(1/711=0.14%) |
| 3. Number of sexual partners in the past 3 months = 0 AND use of alcohol before or during sexual intercourse in the past 3 months: YES | 1(1/711=0.14%) |
| 4. Number of sexual partners in the past 3 months = 0 AND use of substance before or during sexual intercourse in the past 3 months: YES | 0 |
| 5. Number of sexual intercourses in the past 3 months = 0 AND Number of condom use > 0 | 1(1/711=0.14%) |
| 6. Number of sexual intercourses in the past 3 months = 0 AND use of alcohol before or during sexual intercourse in the past 3 months: YES | 1(1/711=0.14%) |
| 7. Number of sexual intercourses in the past 3 months = 0 AND use of substance before or during sexual intercourse in the past 3 months: YES | 0 |
